# Posterior Coronary Vein as the Substrate for an Epicardial Accessory Pathway

**DOI:** 10.1016/s0972-6292(16)30648-9

**Published:** 2013-08-01

**Authors:** Laura Cipoletta, Juan Acosta, Lluis Mont, Antonio Berruezo

**Affiliations:** Arrhythmia Section, Cardiology Department, Thorax Institute, Hospital Clinic, Universitat de Barcelona, Catalonia, Spain

**Keywords:** Coronary sinus myocardial coat, Left posterior accessory pathways, CT imaging, Electro-anatomic mapping, Wolff-Parkinson-White syndrome

## Abstract

Catheter ablation of Wolff-Parkinson-White syndrome is associated with up to 5% of failure. Coronary sinus (CS) abnormalities or connections between CS myocardial coat and left ventricular epicardium are associated with posteroseptal and left posterior accessory pathways (AP). A 41-year-old patient with WPW syndrome was referred to our hospital after three unsuccessful ablations. The 12-lead ECG suggested a left posteroseptal AP. CT imaging and electro-anatomic mapping showed a relationship between AP electrical course and CS posterior branch. This finding supports the hypothesis CSAPs lie in the myocardial coat around CS and represent an extensive connection between atrial and ventricular epicardial surface.

## Introduction

Accessory pathways (APs) causing Wolff-Parkinson-White (WPW) syndrome usually have endocardial ventricular and atrial insertions located close to the atrioventricular (AV) valve rings, making catheter ablation relatively easy and yielding a high success rate. However, this is not the case when the AP is epicardial or its atrial or ventricular insertion is located far from the AV groove [1].

Jackman and colleagues have shown that left-sided APs usually have an oblique course through the mitral annulus and that the ventricular insertion may be located distal to the AV ring [2]. In recent years several authors have proposed that the anatomical substrate of left posteroseptal pathways may be related to the presence of coronary sinus (CS) aneurysms. These diverticula contain myocardial fibers extended from the CS myocardial coat and support the electrical and anatomical connection between left atrium and left ventricle through these aneurysms [3]. However, Sun et al recently reported that up to 70% of these coronary sinus accessory pathways (CSAPs) occur in the absence of CS diverticula or other venous anomaly suggesting the existence of APs formed by connections between extensions of the CS myocardial coat and the epicardial surface of the left ventricle [4].

The unique electroanatomic (EA) characteristics of these AP may not be fully assessed by conventional electrophysiology techniques and fluoroscopy. Three-dimensional (3D) EA mapping has been used to map unusual locations of APs facilitating catheter ablation [5]. Furthermore, in the case of left posteroseptal APs, three-dimensional EA mapping systems offer the possibility of displaying detailed CS anatomy obtained by computed tomography (CT) angiography imported into the mapping system.

We report an illustrative case of an epicardial left posteroseptal AP in which a 3D EA map merged with heart CT was used to delineate the 3D anatomy of the left atrium, left ventricle, CS and cardiac veins, finding correspondence between the registered AP's potentials (at its atrial insertion, central body and ventricular insertion) and the course of a posterior coronary vein.

## Case Report

A 41-year-old male patient with WPW syndrome was referred to our hospital for catheter ablation after three unsuccessful ablation attempts in another hospital. This patient was highly symptomatic, with frequent episodes of orthodromic AV reentrant tachycardia despite antiarrhythmic drugs.

**Figure 1:**
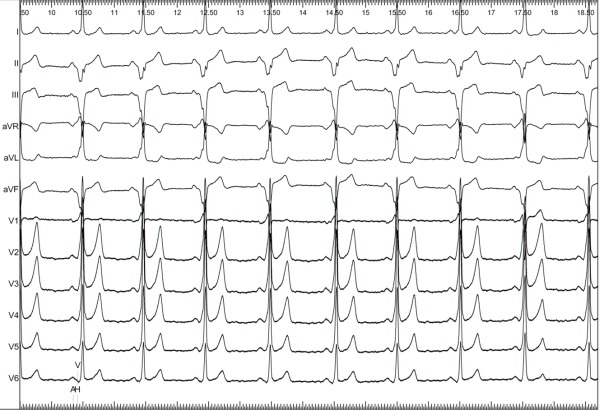
Baseline 12 lead-ECG showing preexcitation with a positive delta wave in V1-V6 and a negative delta wave in II, III, and aVF, with the highest amplitude in leads II and III, suggesting a left posteroseptal AP.

The 12-lead ECG showed preexcitation with a positive delta wave in V1-V6 and a negative delta wave in II, III, and aVF, with the highest amplitude in leads II and III, suggesting a left posteroseptal AP (Figure 1). During previous attempts, radiofrequency (RF) lesions were placed on posteroseptal region of right ventricle (RV), posteroseptal region of left ventricle (LV) and inside CS.

A quadripolar diagnostic catheter and a 3.5 mm cooled-tip RF catheter (ThermoCool NaviStar-RMT, Biosense Webster, Diamond Bar, CA) were advanced through the right femoral vein and placed at right atrium (RA) and His position, respectively. In sinus rhythm the HV interval was 6 ms. The AP effective refractory period could not be estimated because of the induction of preexcitated atrial fibrillation with a single atrial extrastimulus coupled at 250 msec. The shortest R to R interval during atrial fibrillation was 230 msec.

Before ablation, a contrast-enhanced 64-slice CT scan was acquired. These data were loaded into the EA mapping system (CARTO, Biosense Webster, Inc, Diamond Bar, Calif) to provide 3D CT surface reconstruction of the CS, coronary veins, left atrium, RV and LV. Endocardial EA mapping of the RV was performed during sinus rhythm and was merged with the CT scan, allowing real-time visualization of the catheter tip in relation to the cavities, CS veins and epicardium.

**Figure 2:**
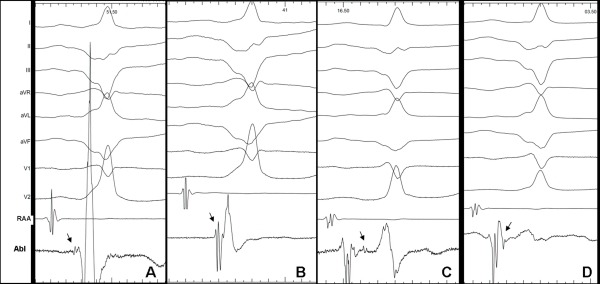
A. Left ventricle (LV) endocardium: low-amplitude Kent potential preceding ventricular electrogram. B. LV epicardium: high-amplitude Kent potential preceding ventricular electrogram. C. Accessory pathway potential isolated from the atrial and ventricular electrograms corresponds to the central body of accessory pathway. D. A Kent potential following the atrial electrogram, corresponding to the accessory pathway's atrial insertion is shown. Black arrows indicate Kent potential.

No significantly premature ventricular activation was observed during RV mapping (10 ms). When mapping inside the proximal CS, early ventricular activation preceded the delta wave by 15 ms. Two focal RF applications at this point failed to abolish preexcitation. The ventricular aspect of posterior mitral annulus was also mapped and a low-amplitude (0.026 mV) AP potential was observed (Figure 2A). This potential was in continuity with the ventricular local electrogram that slightly preceded the delta wave of 12 ms.

The 3-D mapping during RV pacing was performed in the atria to localize the AP atrial insertion. It was located far from the AV groove, without fusion between ventricular and atrial electrogram and RF applications were not effective in suppressing preexcitation.

Percutaneous pericardial access was then obtained using a subxiphoid approach. During mapping of the epicardial LV surface, the earliest ventricular activation was found at the posterobasal segment of the LV at 11.6 mm from the AV groove, matching with the distal end of a posterior vein. Electrograms obtained at this point revealed a ventricular activation that preceded the delta wave by 23 ms and a high-amplitude (0.295 mV) AP potential showing fusion with the ventricular electrogram (Figure 2B). Therefore, this corresponded to an epicardial ventricular insertion of a left posterior AP located relatively far from the mitral annulus. Three RF applications (45ºC; 40 Watts) at this point were not effective.

**Figure 3:**
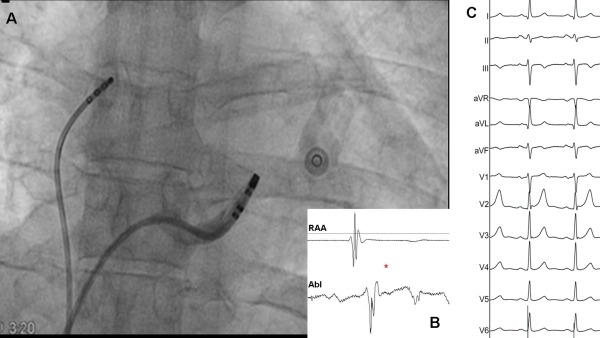
A. Coronary sinus (CS) angiography showing successful ablation site. B. (*) Disappearance of the accessory pathway potential after successful ablation . C. Twelve lead-ECG after ablation showing delta-wave disappearance and narrower QRS.

**Figure 4:**
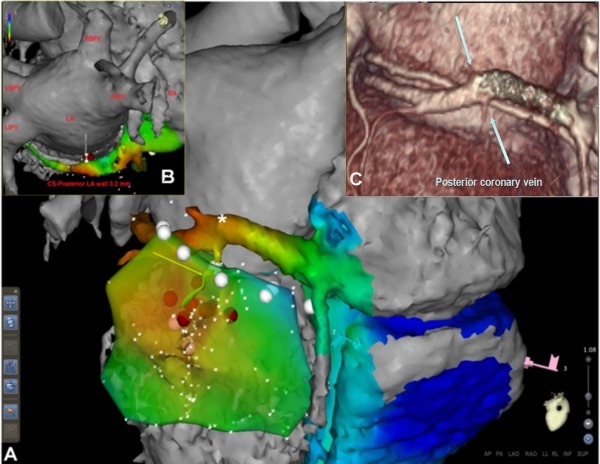
A.- Left ventricle (LV) epicardium and coronary sinus (CS) electroanatomical map merged with computed tomography scan. Red dots represent ablation attempts at the accessory pathway's epicardial ventricular insertion. The yellow arrow highlights the course of a posterior coronary vein from the successful ablation site (*) to the epicardial ventricular insertion site. B.- The successful ablation point inside CS from a cranial-posterior view is shown. C.- 3D reconstruction of CS anatomy obtained from CT scan. (*) indicates the successful ablation site. Light blue arrow highlights a posterior branch of the CS. CS: coronary sinus; LA: left atrium; LSPV: left superior pulmonary vein; LIPV: left inferior pulmonary vein; RSPV: right superior pulmonary vein; RIPV: right inferior pulmonary vein; RA: right atrium.

The CT and CS angiography revealed the presence of a valve inside the CS, which could explain the failure of previous attempts at cannulation of distal CS. A new attempt successfully passed through this anatomic obstacle, allowing distal CS mapping. Once the ablation catheter was advanced 30 mm from CS ostium (Figure 3A), an atrial electrogram was recorded, followed by a Kent potential and a ventricular electrogram without AV fusion (Figure 3C-D). At this site the minimum distance was found between the CS and the posterior wall of the left atrium (3 mm, Figure 4B). In addition to this, the continuity observed between the atrial electrogram and the Kent potential suggested the location of the ablation catheter at the atrial insertion of the AP. RF energy with a target temperature of 45ºC was started at 20 W. A single RF delivery resulted in 2:1 conduction through the AP. A second application successfully abolished preexcitation (Figure 3B and 3C).

A comparison of CT data with the fused endocardial and epicardial EA maps shows the relationship between electrogram location and the anatomic substrate of the AP, from the atrial wall to the CS and following the course of the posterior coronary vein until its insertion distally to the AV groove (Figure 3A and 3C).

## Discussion

Catheter ablation of Wolff-Parkinson-White syndrome (WPW) can be challenging and is associated with failure in up to 5% of cases. Several reviews have evaluated possible reasons. Sacher and co-workers reported that the main causes for initial failure were difficulties in catheter manipulation, inadequate AP mapping primarily due to AP localization (parahisian, epicardial), multiple (>3) pathways, multiple insertions, or congenital heart diseases [1].

Previous studies have demonstrated that CS abnormalities (diverticula, aneurysms) are associated with posteroseptal and left posterior APs. The main cause of these CS anatomic anomalies is the abnormal embryological development of the sinus venosus and the AP results from remnant of the muscle sheath around the proximal CS [3]. Sun and co-workers, supported by previous anatomic findings of Von Ludinghausen, hypothesized that connections between the CS myocardial coat and the LV epicardium could produce an AP even in the absence of a CS anatomic anomaly. These connections could partly explain up to 47% of previous failed ablations of posteroseptal or left posterior APs. Usually the CSAPs have an oblique course because of the oblique orientation of the fibers connecting the CS myocardial coat with the left atrium. Extensive connections also have been found between the CS myocardial coat and the atria, although the localization of the ventricular insertion has not been clearly demonstrated [4].

In the current case three previous attempts had failed to ablate the epicardial posteroseptal AP, despite multiple RF applications delivered at right posteroseptal, left posteroseptal sites and CS. The presence of a negative delta wave in lead II suggested an epicardial localization of the AP. Previous studies indicate about 70% sensitivity of negative delta wave in lead II in identifying a CSAP [4]. Due to the complexity of the AP ablation we decided to use the CARTO system integrated with CT imaging. The CT angiography showed no CS anatomic anomalies but revealed the presence of a valve that could have prevented previous attempts from reaching the distal segment of the CS where the ablation was finally effective.

Electroanatomical mapping integrated with CT imaging obtained an exact reconstruction of CS, its branches and AP potential along its course. The CS mapping obtained an AP potential fused with the atrial potential, clearly representing the AP atrial insertion, with a 55 ms precocity compared to delta wave. Epicardial mapping recorded a high-amplitude (0.295 mV) AP potential fused with a ventricular electrogram. This target point was 1.3 cm from the AV groove and 2.3 cm from CS where the AP atrial insertion was detected. Hence, this feature suggested a large AP course. Only the AP atrial insertion ablation was successful, in a target site localized inside CS at 3 cm from the ostium and at 3 mm from the left atrial posterior wall. Despite the high amplitude of the AP epicardial potential, the ablation was not effective at the ventricular insertion, possibly because of multiple connection sites between the CS musculature and LV epicardial surface or the presence of epicardial fat.

This case is illustrative because CT imaging was performed in addition to EA mapping and showed a relationship between the AP electrical course and a CS posterior branch. This finding could support the hypothesis, postulated by Jackman and co-workers, that CSAPs lie in the myocardial coat around CS and its branches and represent an extensive connection between atrium and ventricular epicardial surface.

## Conclusion

This case illustrates the value of CT imaging integrated with 3D EA mapping in obtaining a reliable anatomic reconstruction of CS and facilitating successful ablation of epicardial posteroseptal APs. The case gives additional support to the hypothesis of Jackman and co-workers about epicardial CSAPs that follow the myocardial coat around the CS and its branches, by showing the tight relationship between AP potential and its anatomic substrate.
